# Simple and Efficient Algorithm for Improving the MDL Estimator of the Number of Sources

**DOI:** 10.3390/s141019477

**Published:** 2014-10-17

**Authors:** Dayan A. Guimarães, Rausley A. A. de Souza

**Affiliations:** National Institute of Telecommunications (Inatel), Av. João de Camargo, 510, Santa Rita do Sapucaí MG 37540-000, Brazil

**Keywords:** AIC, estimation of the number of sources, iMDL, MDL, RMT

## Abstract

We propose a simple algorithm for improving the MDL (minimum description length) estimator of the number of sources of signals impinging on multiple sensors. The algorithm is based on the norms of vectors whose elements are the normalized and nonlinearly scaled eigenvalues of the received signal covariance matrix and the corresponding normalized indexes. Such norms are used to discriminate the largest eigenvalues from the remaining ones, thus allowing for the estimation of the number of sources. The MDL estimate is used as the input data of the algorithm. Numerical results unveil that the so-called norm-based improved MDL (iMDL) algorithm can achieve performances that are better than those achieved by the MDL estimator alone. Comparisons are also made with the well-known AIC (Akaike information criterion) estimator and with a recently-proposed estimator based on the random matrix theory (RMT). It is shown that our algorithm can also outperform the AIC and the RMT-based estimator in some situations.

## 1. Introduction

The estimation of the number of sources of signals impinging on multiple sensors is a fundamental problem in communications and signal processing. This number is important in itself in a huge number of applications [1–9], e.g., to determine the approximate number of neurons responding to some stimulus in the brain [10], to estimate the number of active muscles during an action for identifying the action and determining pathologies [11], to find the number of chemical elements in a mixture [12], to determine the number of speakers in a room with background noise and reverberation [13] or, yet, in a cognitive radio network, to estimate the number of active radio transmitters in a given area [14] or to find the best spectrum opportunities [15]. In other applications, it is used as the input for subsequent estimations, e.g., for some direction of arrival (DoA) estimation and source separation methods in array processing [16,17], for detecting the number of signals in a radar measurement [18] and cognitive radio applications [19] and for estimating the parameters in Markov models [20]. Some DoA estimators do not demand the prior knowledge of the number of sources and apply, as well, to the estimation of this number [7]. Some source separation algorithms also do not need prior knowledge of the number of sources and can also be used for estimating this number [8,9].

In spite of the multitude of techniques available for solving the problem of estimating the number of sources, most of them adopt information theoretic approaches, such as the minimum description length (MDL) and the Akaike information criterion (AIC) [1–5,13,14,21]. The AIC achieves high detection performance (*i.e.*, high probability of correctly estimating the number of sources) at low signal-to-noise ratio (SNR) regimes, but it is not consistent. The MDL estimator is consistent, but degrades its performance at low SNR regimes when compared with the MDL. Nevertheless, over the years, the MDL has been considered a standard when assessing the performance of algorithms for estimating the number of sources [2].

Recently, the random matrix theory (RMT) approach has been proposed to tackle the estimation of the number of source problem [2,22]. In [2], an RMT-based sequential hypothesis test is proposed, claiming high detection performance at low SNR, similar to the AIC estimator, and near consistency at large sample sizes, similar to the MDL estimator. In [22], an improved RMT-based algorithm is proposed. It is also based on a sequential hypothesis test, but the test statistics were derived so as to consider the interaction between signal and noise eigenvalues in the small sample size situation, which is not considered in the original algorithm of [2]. By combining the original algorithm with the new one, improved performance is achieved for both the low and the high SNR regimes.

In this paper, we propose a new algorithm for improving the performance of the MDL estimator. We call it norm-based improved MDL (iMDL). Our algorithm enjoys low complexity, yet exhibits consistency and outperforms the AIC estimator and the RMT-based estimator of [2] in some situations. The performance of the iMDL is always better than or equal to the performance achieved by the MDL estimator alone.

The remainder of the paper is organized as follows. Section 2 presents the problem formulation related to eigenvalue-based source estimators and describes briefly the AIC-, the MDL- and the RMT-based estimators. The proposed iMDL algorithm is described in Section 3. The simulation setup, numerical results and discussions concerning the influence of the system parameters on performance are shown in Section 4. Finally, in Section 5, concluding remarks are drawn and opportunities for future work are highlighted.

## 2. Eigenvalue-Based Estimators

In this paper, we focus on methods that use the eigenvalues of the received signal covariance matrix for estimating the number of sources. We first address the problem formulation and then briefly describe the AIC, the MDL and the RMT-based estimators.

### 2.1. Problem Formulation

Let there be an array with *m* sensors (antennas, for example), each one collecting *n* samples of the received signal from *p* sources (transmitters). These samples are arranged in a matrix **Y** ∈ ℂ^*m*×*n*^, and the samples from the *p* sources are arranged in a matrix **X** ∈ ℂ^*p*×*n*^. Let **H** ∈ ℂ^*m*×*p*^ be the channel matrix with elements {*h_ij_*}, *i* = 1,2,… ,*m* and *j* = 1,2,…, *p*, representing the channel gains between the *j*-th source and the i-th sensor. Finally, let V ∈ C^*m*×*n*^ be a matrix of additive Gaussian noise samples, distributed 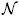 (0, *σ^2^***I***_m_*) and independent of the signal samples. The matrix of received samples is then:

(1) Y=HX+V

In this scenario, we consider only those methods for estimating the number of sources that use the ordered eigenvalues {λ_1_ ≥ λ_2_ ≥ ⋯ λ*_m_*} of the received signal population covariance matrix **R**, for which the maximum likelihood estimate is the sample covariance matrix:

(2) $$\hat{R}=\frac{1}{n}{\mathrm{YY}}^{†}$$

where ^†^ means complex conjugate and transpose. It can be shown [1] that **R** can be written as:

(3) $$R=E[{YY}^{†}]={HR}_{x}H^{†}+\sigma^{2}I$$

where **R***_x_* is the transmitted signal population covariance matrix, **I** is the identity matrix, *σ*^2^ is the noise variance and 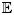
[·]is the expectation operator. If **H** is full column rank, the rank of **HR***_x_***H**^†^ is *p*, which means that the *m* − *p* smallest eigenvalues of **HR***_x_***H**^†^ are equal to zero. Therefore, the *m* − *p* smallest eigenvalues of **R** are equal to *σ*^2^. Then, it is possible to estimate the number of sources *p* from the multiplicity of the smallest eigenvalues of **R**. Instead of **R**, in practice, its estimate **R̂** is computed using a finite number of samples, and the resulting eigenvalues are all different with probability one. In this case, the classification of the eigenvalues in two groups (the largest *p* and the smallest *m* − *p*) is not trivial, representing a challenge for the estimation of the number of sources.

The fundamental difference among the eigenvalue-based methods for estimating the number of sources resides in the way that the eigenvalues are classified. In the following two subsections, we describe three of these methods: the AIC, the MDL and the RMT-based algorithm proposed in [2].

### 2.2. The AIC and the MDL Estimators

The AIC and the MDL estimators have been derived from information theoretic foundations, which renders these estimators the classification as information-theoretic methods [1]. The AIC has been derived from the Kullback–Leibler information concept [23], and the MDL has been derived from a Bayesian approach [24,25]. By departing from different roots, they have ended up in very similar structures; they are also referred to as maximum penalized likelihood methods, for which a compensation or penalization term is subtracted from the likelihood function of the parameters to be estimated. Specifically, the criteria for AIC and MDL are, respectively [1]:

(4) $$\text{AIC}(k)=-2n\ln\frac{\prod_{i=k+1}^{m}\lambda_{i}}{{\left(\frac{1}{m-k}\sum_{i=k+1}^{m}\lambda_{i}\right)}^{m-k}}+2k\;(2m-k)$$

(5) $$\text{MDL}(k)=-n\ln\frac{\prod_{i=k+1}^{m}\lambda_{i}}{{\left(\frac{1}{m-k}\sum_{i=k+1}^{m}\lambda_{i}\right)}^{m-k}}+\frac{1}{2}k\;(2m-k)\ln n$$

for *k* = 0,1, …, m − 1. For both, the estimate *p̂* of the number of sources is the value of *k*, which minimizes the criterion. Notice that, apart from a constant, both estimators have a common first term and different penalization terms. These penalization terms act by compensating, in different ways, the overestimation of the model order (the number of sources), which is known to be produced by the first term [5].

### 2.3. The RMT-Based Estimator

The algorithm proposed in [2] is based on a sequence of hypothesis tests. In each step, the significance of the *k*-th eigenvalue of the covariance matrix of the signal received by *m* sensors is tested. For *k* = 1,2, …, min(*m*, *n*) − 1, it is tested if:

(6) $$\lambda_{k}>{\hat{\sigma }}^{2}(k)C_{n,m,k}(\alpha )$$

where *σ̂*^2^(*k*) is the noise variance estimate on step *k* and *C_n,m,k_* (*α*) is a parameter that depends on the confidence level *α* ≪ 1, as determined by the user. The value of *α* is referred to as the asymptotic false alarm (overestimation) probability.

The computation of *C_n,m,k_* (*α*) involves the calculation of the inverse of a Tracy-Widom distribution, which is a quite difficult process, since this distribution does not have a closed-form expression. Nevertheless, in [26], the authors of [2] provide MATLAB routines capable of inverting numerically the Tracy-Widom distribution, which facilitates the computer implementation of the RMT-based algorithm.

Then, the RMT-based estimate of the number of sources can be written as [2]:

(7) $$\hat{p}=\arg\underset{k}{\min}\left\{\lambda_{k}<{\hat{\sigma }}^{2}(k)C_{n,m,k}(\alpha )\right\}-1$$

Notice that this method suffers from the need of computing a threshold that depends on an estimate of the noise variance, but on the other hand, permits control of the overestimation probability, which can be advantageous in some applications. A noise variance estimation process is also suggested in [2].

## 3. The Proposed iMDL Algorithm

**Figure 1.**
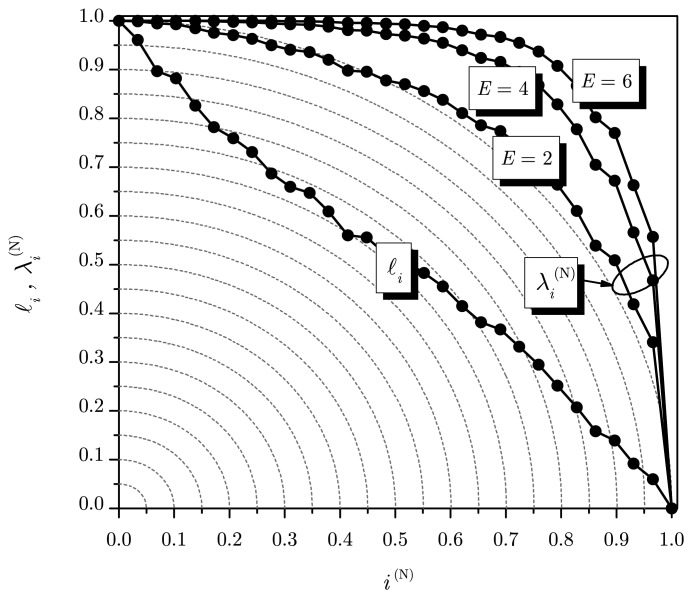
Graphical representation of the normalized eigenvalues and indexes.

Assume that the ordered eigenvalues of **R̂** and the corresponding indexes are normalized, so that both are placed in the interval [0,1], that is, for *i* = 1, …, *m*,

(8a) $$\ell_{i}=\frac{\lambda_{i}-\lambda_{m}}{\lambda_{1}-\lambda_{m}}$$

(8b) $$i^{\left(N\right)}=\frac{i-1}{m-1}$$

(8c) $$\lambda_{i}^{\left(N\right)}=\sqrt{1-{\left(1-\ell_{i}\right)}^{E}}$$

where ℓ*_i_* and *i*^(N)^ are the *i*-th normalized eigenvalues and indexes, respectively. Notice in Equation (8) that the normalized eigenvalues {ℓ*_i_*} are further modified to $\left\{\lambda_{i}^{\left(N\right)}\right\}$ by a nonlinear operation whose role is explained with the help of an example: Figure 1 shows the normalized eigenvalues {ℓ*_i_*} and $\left\{\lambda_{i}^{\left(N\right)}\right\}$ for some values of the exponent *E*, assuming *m* = 30, *p* = 0 and *n* = 5000. Since *p* = 0, as *n* grows, the curve for {ℓ*_i_*} tends to become a straight line. The role of the nonlinear operation on {ℓ*_i_*} is to bend the curve according to the value of *E*. For *E* = 2 and large *n*, the curve for {ℓ*_i_*} tends to lay on the unit semicircumference. For *E* > 2, the curves for $\left\{\lambda_{i}^{\left(N\right)}\right\}$ are further bent.

**Figure 2.**
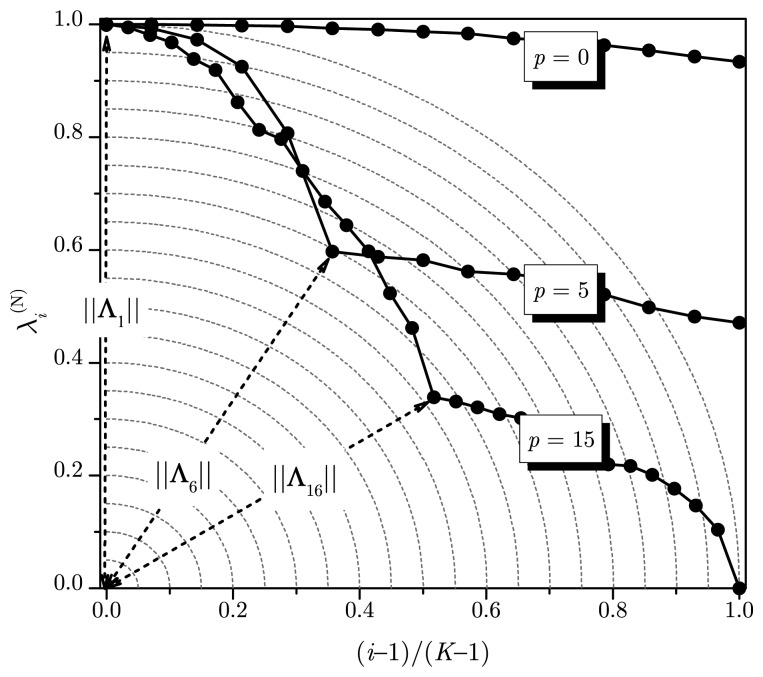
Normalized eigenvalues and indexes according to the iMDL algorithm.

Now, define a vector $\Lambda_{i}={\left[\lambda_{i}^{\left(N\right)}i^{\left(N\right)}\right]}^{T}.$. Notice in Figure 1 that, for *E* > 2, ‖**Λ**_1_‖ < ‖**Λ***_i_*‖ for *i* ≠ 1, where ‖**Λ***_i_*‖ is the Euclidean norm of **Λ***_i_*. For *p* > 0, the smallest *m* − *p* eigenvalues of **R̂** tend to be equal to the noise variance, and an inflection point in the curve will become evident, at the transition from the largest *p* and the smallest *m* − *p* eigenvalues, as illustrated in Figure 2. This will tend to make ‖**Λ***_p_*_+1_‖ < ‖**Λ***_i_*‖ for *i* ≠ *p* + 1. Then, ‖**Λ***_i_*‖ can be used to estimate the number of sources.

For a small number of sources, however, the placement of the inflection point more to the left will lead to a high chance of having ‖**Λ***_i_*‖ < ‖**Λ***_p_*_+1_‖ for *i* > *p* + 1. To avoid this, only a subset of vectors of the set {**Λ***_i_*} should be tested while searching for the smallest norm. Figure 2 illustrates this heuristic for a subset size *K* = 15, with *p* = 0 and *p* = 5, assuming *m* = 30, *n* = 5000 and *E* = 5. Notice that searching within a subset of a size smaller than *m* is equivalent to pushing the inflection point of the curves to the right, reducing the chance of overestimating the number of sources. If the number of sources increases, the entire set with *K* = *m* vectors is used; see an example for *p* = 15 in Figure 2. This heuristic is considered in our algorithm by using the MDL estimate as the reference: we set *K* = m if the MDL estimate is greater than *m*/3 and set *K* = min {⌊*m*/3⌋, 3(*p̂*_MDL_ + 1)} otherwise. The reasoning behind this heuristic goes as follows: During the development of our algorithm, we realized that the value of *K* that maximizes the average probability of correct detection, measured over several different sets of system parameters, could not be *K* = m for any number of sources *p*. Then, through exhaustive search based on the probability of correct detection, we have empirically found that two values of *K* would suffice: *K* = *m*/3 for small values of *p* and *K* = *m* for larger values of *p*. The reference to distinguish between small values and larger ones has been empirically set to *m*/3 (also through exhaustive search). However, if the number of sensors *m* is large and the MDL estimate is by far smaller than *m*/3, the subset size had to be set smaller than *m*/3 to increase the probability of correct detection for low values of *p*. This is the reason for choosing *K* = min {⌊*m*/3⌋, 3(*p̂*_MDL_ + 1)}, where *p̂*_MDL_ is the initial estimate of *p* in our algorithm, obtained from Equation (5).

The iMDL algorithm is summarized in Algorithm 1.

The choice of the bending exponent *E* in Equation (8) can be made via simulation, aiming at maximizing the probability of correctly estimating the number of sources, *P*_c_, for a specific set of system parameters. In a scenario of more practical significance, *E*, can be found as the value that maximizes the average *P*_c_ over several sets of parameters. For example, combining the parameters *m* = 10, 15, 20, 50; *n* = 50, 100, 200, 500, 1000, 50, 000; SNR = −5 dB, 0 dB, 5 dB, 8 dB, 10 dB; *p* = 2,5, 10, 15, the optimal exponent *E* = 5 was found. The numerical results shown hereafter consider *E* = 5.



**Algorithm 1** The proposed iMDL algorithm.
 Compute *p̂*_MDL_ using (5) **if**
*p̂*_MDL_ > *m*/3 **then**  *K* = *m* **else**  *K* = min { ⌊*m*/3⌋, 3(*p̂*_MDL_ + 1)} **end if** **for**
*i* = 1…*m*
**do**  Compute 
$\lambda_{i}^{\left(N\right)}$ using (8) **end for** **for**
*j* = 1…*K*
**do**  Compute 
$\Lambda_{j}={\left[\lambda_{i}^{\left(N\right)}\frac{j-1}{K-1}\right]}^{T}$ **end for** Compute 
$\hat{p}=\max\left\{{\hat{p}}_{\mathrm{MDL}},\arg\underset{j}{\min}\left\|\Lambda_{j}\right\|-1\right\}$


## 4. Simulation Setup and Numerical Results

In this section, we present the simulation setup and numerical results comparing the iMDL, the MDL, the AIC and the RMT-based estimators. Each point in the subsequent graphs was generated from 5000 Monte Carlo events. In each event, a new matrix **Y** = **HX** + **V** was generated. The entries in **X** are independent and identically distributed (i.i.d.) complex Gaussian variates that simulate Gaussian-distributed and uncorrelated transmitted signals. This situation arises, for example, in wireless communications, where the envelope of most modulated signals are Gaussian-distributed, for instance the amplitude of a multi-carrier signal, such as orthogonal frequency-division multiplexing (OFDM), with a large number of subcarriers, which is the preferred modulation technique in most modern wireless technologies, including several digital television standards. The entries in **H** are also i.i.d. complex Gaussian, to simulate a slow flat Rayleigh fading channel, which is constant during each detection interval, changing independently from one interval to the next. The entries in **V** are also i.i.d. complex Gaussian, representing the additive thermal noise present at the receiver inputs. Assuming unitary total transmit power, the received SNR is given by tr[**H**^†^**H**]/ (*mpσ*^2^), where tr [·] is the trace of the underlying matrix. To simulate noise uncertainty in the noise variance estimate for the RMT-based algorithm, the noise variance is made a uniform random variable in [*σ*^2^/1.05,1.05*σ*^2^] = [*σ*^2^/*ρ*, *ρσ*^2^], where *ρ* = 10*^μ^*/^10^ and *μ* is the degree of noise uncertainty, in dB. This situation is denoted by RMT2 in the graphs. Notice that this degree of uncertainty corresponds to *μ* ≈ 0.2 dB, a value that can be considered realistic from a practical standpoint, since typical worst-case values of *μ* are on the order of 1 dB or even more [27].

In order to simulate signal sources with variable power, we assume that the signal strengths are uniform random variables in [0.4,1]. Additionally, for the RMT-based estimator, the asymptotic false alarm (overestimation) probability is adjusted to *α* = 0.1%. In all figures, the probability of correctly estimating the number of sources is denoted by *P*_c_, that is, *P*_c_ = Pr{*p̂* = *p*}, where Pr{·} is the probability of a given event. This probability is usually referred to as the probability of detection in the related literature. Similarly, the probability of overestimating the number of sources is denoted by *P*_oe_, that is, *P*_oe_ = Pr{*p̂* > *p*}. These probabilities were considered as the performance measurements of the estimators. In our simulations, *P*_c_ has been estimated as the ratio between the number of correct detections concerning the number of sources and the total number of test events, in our case 5,000 events for each point in the graphs. In a similar manner, *P*_oe_ has been estimated in our simulations as the the ratio between the number of overestimated detections about the number of sources and the total number of test events.

**Figure 3.**
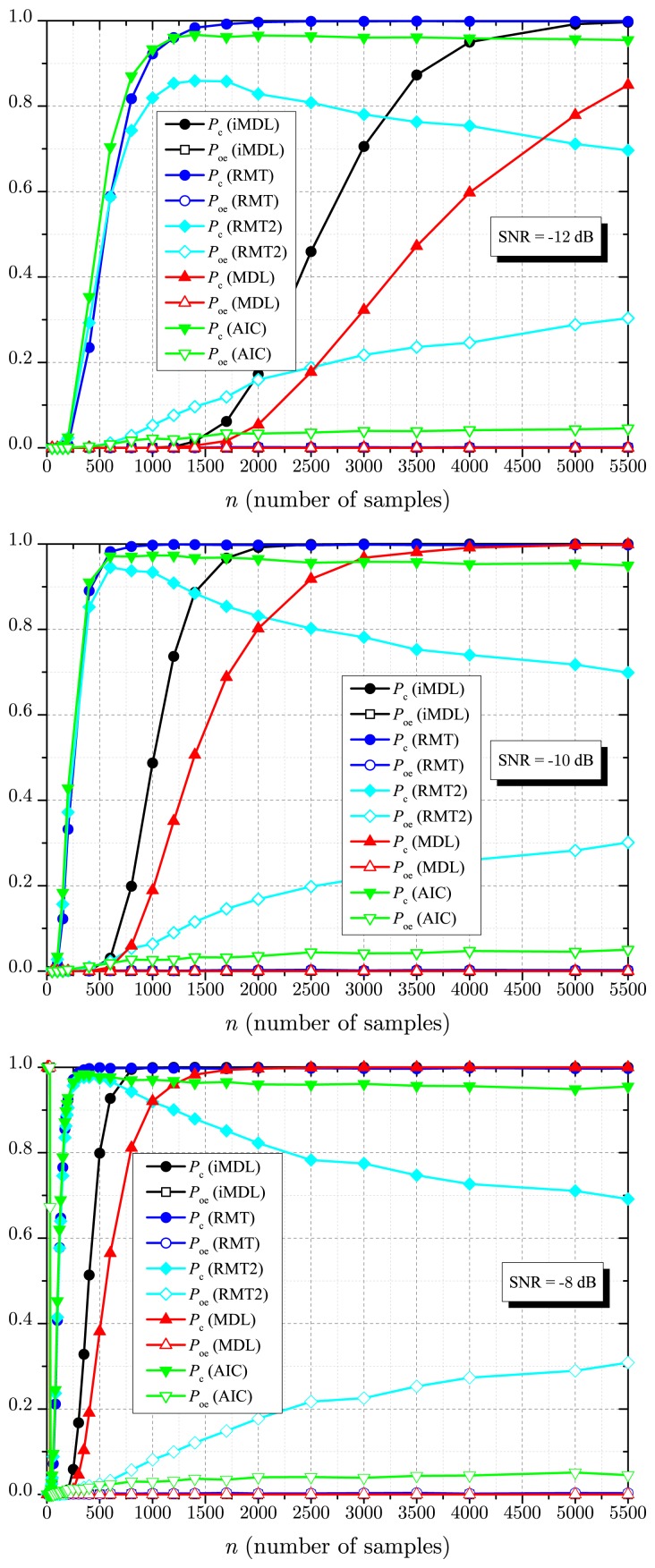
*P*_c_ and *P*_oe_ against *n* for *p* = 3, *m* = 30, SNR = −12 dB (**top**); SNR = −10 dB (**middle**) and SNR = −8 dB (**bottom**).

Figure 3 shows results for *P*_c_ and *P*_oe_ as a function of the number of samples *n* collected by each sensor, for *m* = 30 sensors, *p* = 3 signals sources, SNR = −12 dB, SNR = −10 dB and SNR = −8 dB. Notice that, with perfectly known noise variance, the RMT-based estimator achieves high detection performance at low SNR, similar to the AIC, and near consistency at large sample sizes, similar to the MDL (which is known to be consistent). However, the RMT-based estimator unveils severe inconsistency at large sample sizes in the presence of a small noise uncertainty *μ* = 0.2 dB (RMT2 curves). The AIC estimator reaches higher detection performance with a smaller number of samples, but clearly, it is inconsistent at large sample sizes, having a non-negligible *P*_oe_ for *n* ≫ 1. Notice that the proposed iMDL estimator performs better than the MDL estimator for all considered values of *n* and SNR. It also clearly appears to be a consistent estimator, though we are not able to prove it due to difficult mathematical tractability. Notice also that an increase in the SNR reduces the number of samples necessary for a target *P*_c_, which is a characteristic of all estimators. For example, the number of samples necessary for a target *P*_c_ ≈ 0.7 in the iMDL estimator decreases from 3000 (SNR = −12 dB) to 1200 (SNR = −10 dB) and to 480 (SNR = −8 dB).

**Figure 4.**
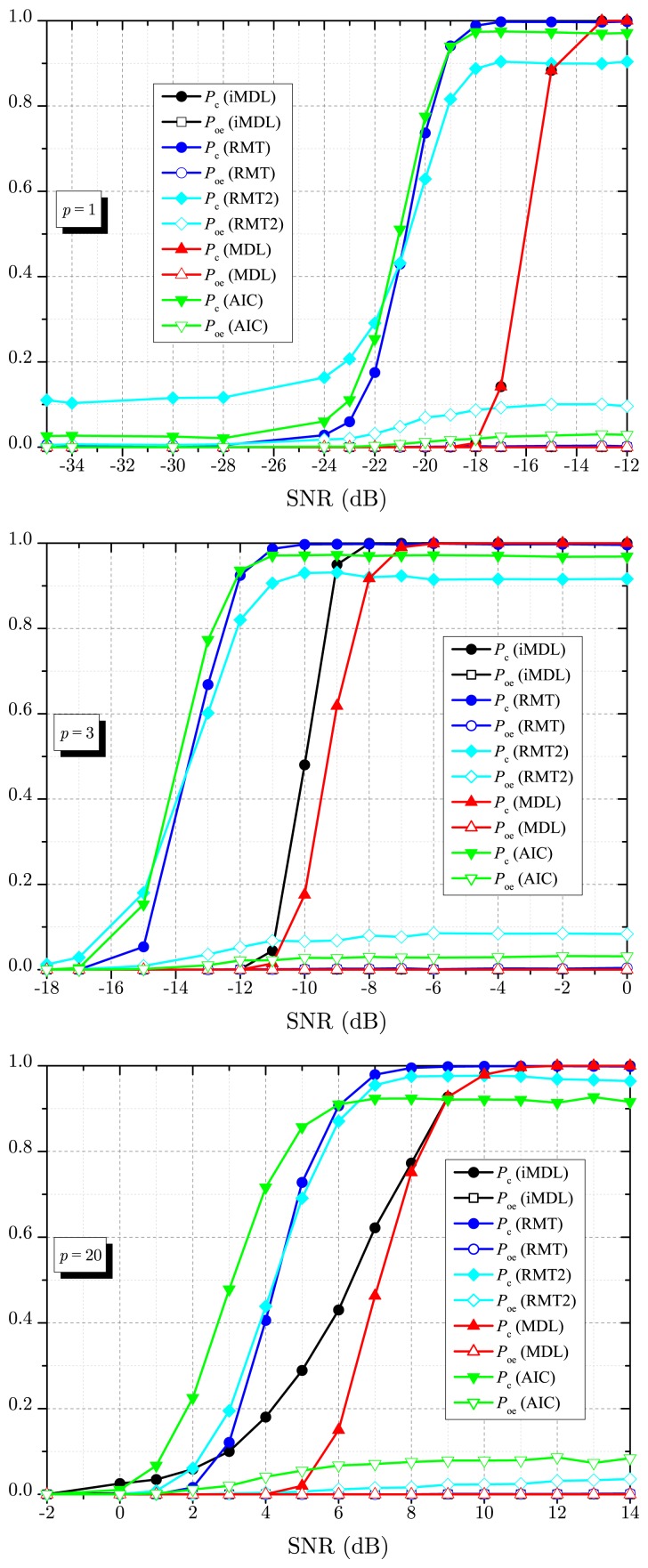
*P*_c_ and *P*_oe_ against SNR for *m* = 30, *n* = 1000, *p* = 1 (**top**); *p* = 3 (**middle**) and *p* = 20 (**bottom**).

Figure 4 depicts numerical results for *P*_c_ and *P*_oe_ as a function of the SNR, for *p* = 1, *p* = 3 and *p* = 20 signals sources, *m* = 30 sensors and *n* = 1000 samples. Again, this figure shows the superior detection capability of the iMDL algorithm when compared with the MDL estimator. Notice that the MDL estimator reaches the same performance of the proposed iMDL algorithm only for *p* = 1. However, this is not meant to state that, if *p* = 1, the MDL and the iMDL estimators will always achieve the same performance, since this depends on the remaining system parameters.

The advantages and drawbacks of the estimators as perceived from Figure 3 are also observable in Figure 4. Particularly, the MDL and the iMDL achieve *P*_c_ = 1 for high values of SNR. The RMT-based estimator achieves *P*_c_ ≈ 1 in this situation; in fact, it achieves *P*_c_ ≈ 1 − *α.* However, when there is uncertainty in the noise variance (RMT2 curves), which reflects a practical scenario, the RMT-based estimator produces a non-negligible *P*_oe_, preventing it from achieving *P*_c_ ≈ 1. Due to inconsistency, the AIC also produces a non-negligible *P*_oe_, leading to *P*_c_ → (1 − *P*_oe_) as SNR → ∞.

**Figure 5.**
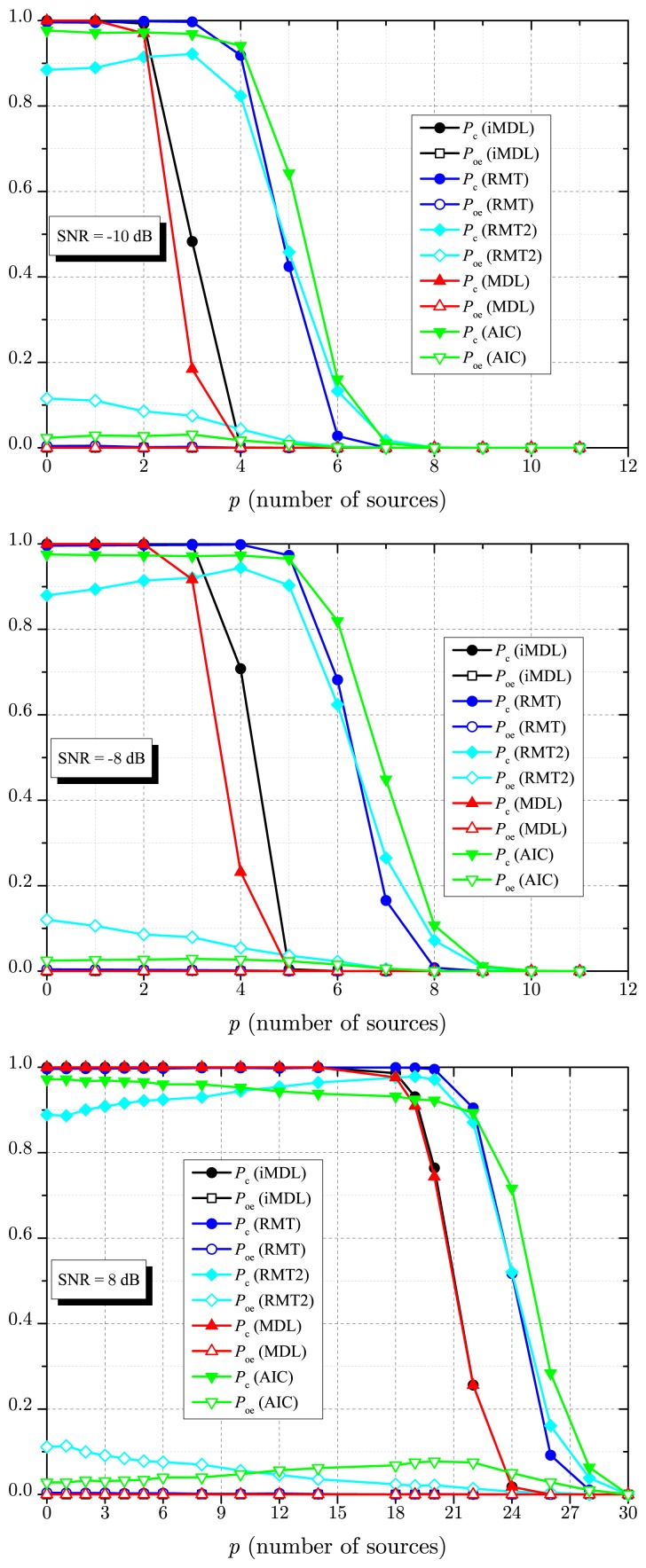
*P*_c_ and *P*_oe_ against *p* for *m* = 30, *n* = 1000, SNR = −10 dB (**top**); SNR = −8 dB (**middle**) and SNR = 8 dB (**bottom**).

Figure 5 shows results for *P*_c_ and P_oe_ as a function of the number of sources *p*, considering *m* = 30 sensors, *n* = 1000 samples, SNR = −10 dB, SNR = −8 dB and SNR = 8 dB. Similar to the previous results, Figure 5 unveils the superior performance of the iMDL when compared with the MDL, mainly in the low SNR regime, *i.e.*, for SNR = −10 dB and SNR = −8 dB. The iMD4L and the MDL performances are almost the same in the high SNR regime, *i.e.*, for SNR = 8 dB. Similarly to the case of *p* = 1 in Figure 4, this is not meant to state that, if the SNR is high, the MDL and the iMDL estimators will always achieve the same performance, for it depends on the remaining system parameters.

Still referring to Figure 5, for a smaller number of sources, both the MDL and the iMDL achieve *P*_c_ = 1. In this situation, the RMT-based estimator achieves *P*_c_ ≈ 1 when the noise variance is assumed to be perfectly known, but its performance is degraded if noise uncertainty takes place (RMT2 curves). The maximum estimated number of sources with high detection probability clearly varies from one estimator to another and with the SNR, with the advantage of the RMT and the AIC. We can also see in Figure 5 that, as the SNR increases, the relative differences among these maximum numbers of sources diminish.

**Figure 6.**
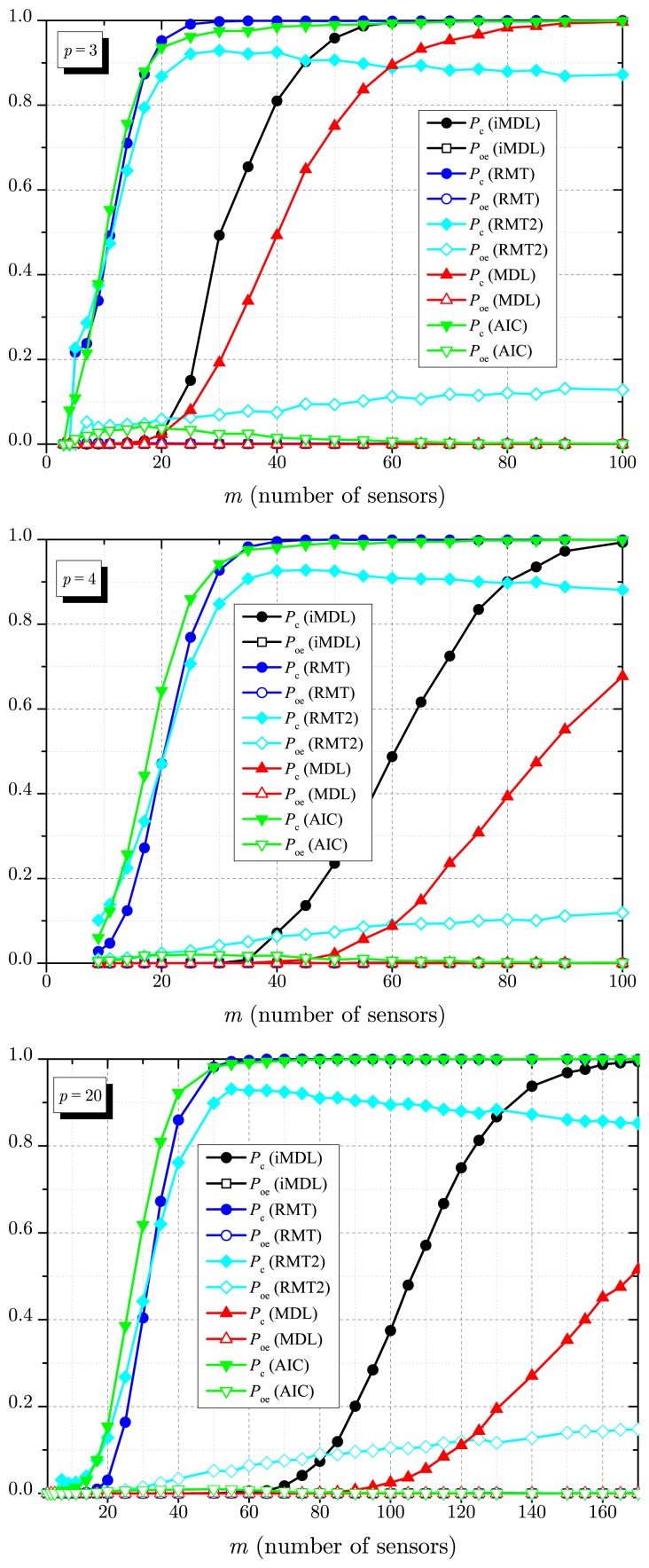
*P*_c_ and *P*_oe_ against *m* for SNR = −10 dB, *n* = 1000, *p* = 3 (**top**); *p* = 4 (**middle**) and *p* = 10 (**bottom**).

In Figure 6, the performance of all estimators are plotted as a function of the number of sensors *m*, considering *n* = 1000 samples, SNR = −10 dB, *p* = 3, *p* = 4 and *p* = 10 signals sources. One more time, we can see the superiority of the iMDL when compared with the MDL, especially for *p* = 10, *i.e.*, with a large number of signal sources. The advantages and drawbacks of the AIC and the RMT-based estimators can again be observed, now with variable *m*.

All of the results reported here considered sets of different values of the main system parameters, namely the number of sources *p*, the number of sensors *m*, the number of samples *n*, the signal-to-noise ratio SNR and the noise uncertainty *μ*. Obviously, different values of these parameters can lead to different performances. As a consequence, a myriad of different scenarios may be exercised. We attest, however, that, for a bunch of other parameters different from those adopted in this paper, we still have verified the superiority of the iMDL against the MDL estimator or at least an equal performance in some few cases.

## 5. Conclusions

This paper proposed an empirical algorithm for improving the performance of the MDL estimator of the number of sources of signals impinging on multiple sensors. The so-called improved MDL (iMDL) uses the MDL estimate as the input data of the algorithm. As shown by the numerical results, the proposed iMDL algorithm achieves performances that are better than or equal to those achieved by the MDL estimator alone. In some situations, the proposed algorithm outperforms the well-known AIC and the RMT-based estimators. Additionally, in contrast to the AIC and to the RMT-based estimator in the presence of noise variance uncertainty, the iMDL is shown to be consistent, though this has not been supported by a mathematical proof. Furthermore, being an empirical proposition, the iMDL is not guaranteed to be superior to the MDL in all possible scenarios, though it has been shown to be indeed superior in a large number of other situations not shown here. We attest that none of these situations has produced an iMDL performance worse than the MDL performance. Last, but not least, it is worth mentioning that our results can be easily reproduced due to the low complexity of the iMDL algorithm.

Besides wireless communications, scenarios that are more applicable to radar and sonar applications could be investigated, as well. They were put aside due to the limited space, but represent important opportunities for further studies and, hopefully, to broaden the applicability of the proposed algorithm.

The continuity of the work suggests comparing the iMDL, proposed in this paper, with the algorithm proposed in [28] with respect to the robustness against the deviation from the spatially white noise assumption of the original MDL and including the new RMT-based estimator of [22] in the comparisons. Additionally, from an implementation point of view, it will be important to perform measurements with real signals using a test-bed with different types of the sources.
